# Air Pollution and Emergency Department Visits for Otitis Media: A Case-Crossover Study in Edmonton, Canada

**DOI:** 10.1289/ehp.0901675

**Published:** 2010-07-21

**Authors:** Roger Zemek, Mieczysław Szyszkowicz, Brian H. Rowe

**Affiliations:** 1 Children’s Hospital of Eastern Ontario and University of Ottawa, Ottawa, Ontario, Canada; 2 Population Studies Division, Health Canada, Ottawa, Ontario, Canada; 3 Department of Emergency Medicine and; 4 School of Public Health, University of Alberta, Edmonton, Alberta, Canada

**Keywords:** air pollution, otitis media, urban

## Abstract

**Background:**

Otitis media (OM) is one of the most common early childhood infections, resulting in an enormous economic burden to the health care system through unscheduled doctor visits and antibiotic prescriptions.

**Objectives:**

The objective of this study was to investigate the potential association between ambient air pollution exposure and emergency department (ED) visits for OM.

**Materials and Methods:**

Ten years of ED data were obtained from Edmonton, Alberta, Canada, and linked to levels of air pollution: carbon monoxide (CO), nitrogen dioxide (NO_2_), ozone (O_3_), sulfur dioxide, and particulate matter (PM) of median aerometric diameter ≤ 10 and 2.5 μm (PM_10_ and PM_2.5_ respectively). A time-stratified case-crossover technique was applied to analyze the associations between ambient air pollution and health outcomes. Conditional logistic regression analysis with the subject’s identification number as a stratum variable was used to obtain odds ratios (ORs) and their corresponding 95% confidence intervals after adjustment for meteorological factors.

**Results:**

We based the analysis on 14,527 ED visits for OM over 10 years in children 1–3 years of age. We observed statistically significant positive associations between ED visits for OM and interquartile increases in CO and NO_2_ levels after adjusting for ambient temperature and relative humidity. We observed the strongest associations (expressed by ORs) in the warmer months (April–September) in girls and all patients for exposure to CO and NO_2_, and in boys for exposure to CO, for 2 days before an OM ED visit.

**Conclusions:**

These results support the hypothesis that ED visits for OM are associated with ambient air pollution.

Otitis media (OM) is one of the most common early childhood infections, resulting in an enormous burden to the health care system through unscheduled doctor visits (Freid et al. 1998) and antibiotic prescriptions (Bondy et al. 2000), resulting in an estimated annual cost of $3 billion to $5 billion annually in the United States (Schwartz and Gates 2003). OM is a multifactorial disease: an immune response in the context of a microbial illness—typically an upper respiratory tract infection (Vergison 2008). Numerous factors can influence the microbial load. A growing body of evidence links OM to ambient air pollutants. Animal studies have demonstrated impairment of ciliary function and increased middle ear mucus secretion after sulfur dioxide (SO_2_) exposure (Ohashi et al. 1989b). Consequently, the possible link between air pollution and OM is important to understand.

Several epidemiologic studies have examined the relationship between ambient air pollution and OM in children, each investigating approximately 300 children (Caceres Udina et al. 2004; Dostal et al. 2001; Holtby et al. 1997; Karmaus et al. 2001; Ribeiro and Cardoso 2003). Dostal et al. (2001) used a cross-sectional study of 452 children 0–3 years of age comparing two cities with high and low ambient air pollution in the Czech Republic [particulate matter (PM) and SO_2_]. Caceres Udina et al. (2004) studied a prospective cohort study of 229 newborns in Spain living in areas of differing pollution. Holtby et al. (1997) examined school children in the United Kingdom in relation to the distance between an industrial emission sources at the home address in a cross-sectional study. Ribeiro and Cardoso (2003) repeated cross-sectional studies of three Brazilian neighborhoods of São Paulo in 393 children (SO_2_ and PM). Karmaus et al. (2001) used a cross-sectional study of 343 children in Germany, looking at organochlorine exposure in the blood as a marker for exposure to emissions. Despite methodological differences, each study demonstrated associations between otitis and exposure to ambient air pollution. Heinrich et al. (2002) over 8 years repeated three cross-sectional studies of 7,000 children with an embedded cohort living in two polluted areas and one control area in East Germany and found that when air quality improved, the prevalence of OM decreased.

Brauer et al. (2006) recently published data from two prospective birth cohorts of approximately 4,350 infants in the Netherlands and Germany in which ambient air pollutants were associated with OM. Outside Europe, however, there is a paucity of data examining this potential link (Brauer et al. 2006). The only published North American study examining emergency visits and air pollution was conducted in Prince George, British Columbia, Canada, and found associations with respiratory illnesses but did not report on OM (Jorgensen et al. 1996). A recent comprehensive review of published evidence, however, was unable to confirm that ambient air pollution is a risk factor for OM in children (Heinrich and Raghuyamshi 2004) and concluded that the current data were too sparse. Thus, whether an association truly exists between ambient air pollution and OM remains an unanswered question.

In this research, we applied a time-stratified case-crossover study design to estimate effects of ambient air pollution on emergency department (ED) visits for OM in Edmonton, Alberta, Canada, in children. In addition, one of the major limitations of previous studies in this field is the close link between socioeconomic status and air pollution exposures. The case-crossover methodology used in our study, in which each child serves as his or her own control, reduces or eliminates confounding by factors associated with crowded living conditions and air pollution exposures. We considered ambient air pollution and weather as an exposure and ED visits for OM as a health outcome. As with other similar research, our hypothesis was that the likelihood of a visit to the ED for OM would increase as ambient air pollution concentrations increased, if exposure to outdoor levels of air pollution is an important risk factor for OM.

## Materials and Methods

### Study design

For our study, we used a case-crossover design that is beneficial for studying relationships that have short time intervals for individual exposure, disease process, and induction periods (Jaakkola 2003; Janes et al. 2005; Maclure 1991). This model has been preferred in previous air pollution studies because it also allows for the case individuals to become their own controls.

### ED visit

ED visit data were obtained from five different hospitals in the greater Edmonton region over a 10-year period (1 April 1992–31 March 2002). These hospitals each have an annual ED visit census ranging from approximately 45,000 to 60,000 patients. One hospital is a tertiary children’s hospital linked to an adult university hospital, one is an academic hospital ED with mixed adult and pediatric populations, and three were community-based EDs with mixed pediatric and adult patient populations.

### OM cases

ED visits for OM were identified based on a discharge diagnosis of OM using the *International Classification for Diseases, 9th revision* (ICD-9; World Health Organization 1975), rubric (code 382.9 only) for children 1–3 years of age. We did not distinguish between subtypes of OM (e.g., with and without effusion) because > 99% of all the discharge diagnoses from the ED in children 1–3 years of age stated only “otitis media.” Our age constraint includes the age with the peak incidence of OM (1–2 years of age) (Daly and Giebink 2000). The visits were date-tagged at the day of arrival to the ED.

### Air pollution measurements

Air pollution data were obtained from the National Air Pollution Surveillance (NAPS) system (http://www.etc-cte.ec.gc.ca/NapsData) using urban background fixed monitors with three stations (northwest, central, and east) covering the city. The largest distance between monitors is about 10 km. [For a map of Edmonton displaying station locations and for further information regarding air pollution in Edmonton and the stations, see Myrick (1996).]

We obtained data on carbon monoxide (CO), nitrogen dioxide (NO_2_), ozone (O_3_), and SO_2_. CO, NO_2_, O_3_, and SO_2_ were measured using “reference methods” or “equivalent methods” as designated by thebU.S. Environmental Protection Agency (2008b). CO was measured using nondispersive infrared spectrometry, NO_2_ using chemiluminescence, O_3_ using chemiluminescence/ultraviolet photometry, and SO_2_ using coulometry/ultraviolet fluorescence. PM with median aerometric diameter ≤ 2.5 and ≤ 10 μm (PM_2.5_ and PM_10_, respectively) was measured using tapered element oscillating microbalance instruments (see NAPS Web site: http://www.etc-cte.ec.gc.ca/NapsData). CO, NO_2_, O_3_, and PM_2.5_ were measured by three stations, PM_10_ by two stations, and SO_2_ by one station. The measurements for PM_10_ were available for January–December 1994 and from March 1995 to the end of the study, March 2002; and for PM_2.5_, from April 1998 to the end of study. When data were available from more than one monitoring station, they were averaged. We did not include days in which more than six of the 24 hourly measurements (one-quarter of hourly values) were missing for the considered air pollutant.

Gasoline and diesel motor vehicles produce CO, NO_2_, and PM (U.S. Environmental Protection Agency 2008a). O_3_ is produced as a by-product of these pollutants and is considered a long-range pollutant. SO_2_ is typically produced from the burning of coal and other sulfur-containing fossil fuels. The daily means were calculated as the average of 24 hourly measures in the same day. Daily data were averaged across the three monitoring stations that were in operation during the study interval. Daily means were used to represent shared exposure of the population in the study.

Weather data were obtained from Environment Canada’s weather archive (http://climate.weatheroffice.gc.ca/). Environment Canada supplied hourly data for relative humidity and temperature (dry bulb) for the City of Edmonton. We calculated the daily levels of temperature and relative humidity by averaging hourly readings (24 measurements) over 24-hr periods. In the final conditional logistic regression models, the weather variables were treated as confounders.

### Statistical analyses

A *p*-value of 0.05 was considered significant in all presented statistical analyses. The associations between ambient air pollution and ED visits for OM were analyzed using a time stratified case-crossover approach (Janes et al. 2005; Maclure 1991). This technique is an adaptation of the case–control study that has cases serve as their own controls, so that individual time-independent confounding factors are controlled by the study design. The case period for each ED visit for OM was defined as the day of presentation. Control periods were selected by matching the same day of the week within the same calendar month and year as the case interval. As a consequence, three or four control periods were determined with irregular occurrences before or after the visit. This approach allows unbiased conditional logistic regression estimates (Janes et al. 2005). We summed all cases of OM across the five hospitals, analyzing the data as though they came from one large hospital.

In our analysis we defined same-day exposure as lag 0 and examined daily exposures for up to 4 days before the ED visit (1- through 4-day lagged exposure). We conducted analysis for the whole period (January–December) and stratified analysis for the warm (April–September) and cold (October–March) months of the year. A stratified analysis was also performed for males and females because of the higher risk (by multivariate analysis) for OM in males (Teele et al. 1989). Also, there is limited evidence to suggest that males might be more susceptible than females to air pollution (Granados-Canal et al. 2005).

Our relative risk estimates were derived primarily by using single-pollutant conditional logistic regression models. The independent variables in the constructed models were air pollution, temperature, and relative humidity represented as continuous variables and were lagged for the same number of days. Results are reported as odds ratios (ORs) and 95% confidence intervals (CIs) for ED visits for OM in association with an interquartile range (IQR; 75th minus 25th percentile values; Table 1) increase in the concentration of the considered air pollutants, adjusted for temperature and humidity.

We recognize the potential for confounding by other air pollutants when focusing on a single variable. We therefore performed multipollutant model to control for confounding and elaboration of the role of residual confounding. Neither single-pollutant nor multipollutant models identified a significant effect of SO_2_ on OM. Therefore, analysis focused on possible mutual confounding among CO, NO_2_, O_3_, and PM.

We also addressed the subject of possible effects of cumulative exposures, testing exposure doses accumulating over few days. Dose was defined as average level of a pollutant on 2–5 successive days preceding the ED visit.

In addition, we estimated associations with first ED visits only, where these visits were classified for uniquely identified patients, with no missing identification number, registered for the first time at the considered age range of 1–3 years. The Supplemental Material (doi:10.1289/ehp.0901675) presents the results for first visits and cumulative and multiple exposures.

### Ethics

The University of Alberta Health Research Ethics Board overseeing the participating hospitals approved the access to data, and the data were transferred to the Health Canada team after deidentification. No patient contact was made, and patients could not be traced.

## Results

We based the analysis on a total of 14,527 ED visits for OM in children 1–3 years of age over a span of 10 years. More ED cases of OM were diagnosed in the cold season (8,706) than in the warm season (5,821), and there were more visits for girls (8,055) than for boys (6,472).

For air pollution measurements, the correlation between air pollution levels for three monitoring stations [northwest (N), central (C), and east (E)] were as follows: for CO, E–N, 0.79; E–C, 0.78; N–C, 0.83, based on 3,512 daily averages; for NO_2_, E–N, 0.74; E–C, 0.80; N–C, 0.78, based on 3,431 daily averages; for O_3_, E–N, 0.85; E–C, 0.89; N–C, 0.88, based on 3,552 daily averages. We obtained SO_2_ measurements from one monitor (E) for 3,652 days (Myrick 1996). PM_10_ was available for 2,813 days, including 835 days with two monitors (N–C, 0.81), and PM_2.5_ was available for 1,440 days (E–N, 0.79; E–C, 0.87; N–C, 0.81, including 506 days with three monitors). Table 1 displays the mean (warm, cold, and all months), SD, and quartiles (25th, 75th percentile values, and IQR, respectively) of daily average concentrations of the ambient air pollutants (CO, NO_2_, SO_2_, O_3_) and meteorologic factors. In general, the mean pollutant levels were higher in the cold seasons than in the warm seasons for CO, NO_2_, and SO_2_, and opposite trend occurred for O_3_ and PM (Table 1).

We observed significant associations between IQR increases in some of the ambient air pollutants and ED visits for OM in all children combined (Table 2). An IQR increase in CO was associated with ED visits for OM in children 1–3 years of age with a 2-day lag (OR = 1.03; 95% CI, 1.00–1.05). This association was stronger in the warm months (OR = 1.14; 95% CI, 1.06–1.23). We observed significant associations with CO lags of 1–3 days in warm months (OR = 1.08; 95% CI, 1.00–1.17 and OR = 1.08; 95% CI, 1.00–1.16, respectively). We found no significant associations between CO and ED visits for OM in cold months in all patients combined.

We observed similar results for IQR increases in NO_2_ and ED visits for OM (Table 2), with significant associations after a 2-day lag for all year and for cold and warm seasons (all seasons: OR = 1.05; 95% CI, 1.01–1.08). We found a stronger association during warm weather months (OR = 1.10; 95% CI, 1.02–1.l9). We also observed significant NO_2_ associations with a 3-day lag in warm seasons. Because we obtained significant relations for both CO and NO_2_, we calculated Pearson correlation coefficients to better understand the associations between two pollutants. NO_2_ and CO were strongly correlated with each other (*r* = 0.47, 0.62, and 0.74 for all, warm, and cold months, respectively). We also observed significant associations between IQR increases in PM_10_ and OM with both 2- and 4-day lags during warm seasons. The association with 3-day lag was quite similar but not statistically significant.

Lags of 1, 2, and 4 days for CO were significantly associated with OM ED visits during warm months when analyses were restricted to males (Table 3). The OR for a 3-day lag also suggested a positive association but was not statistically significant. In cold seasons, CO, NO_2_, and SO_2_ were not significantly associated with ED visits for OM in males. For the long-range O_3_ pollutant, in boys IQR increases in same-day concentrations were significantly associated with ED visits for OM in cold, warm, and all seasons and with an IQR increase in O_3_ with a 1-day lag in cold seasons. We observed no significant associations between PM and OM in boys. However, associations with PM_10_ were comparable with those estimated for all children but were not significant, probably because they were based on fewer observations.

An IQR increase in CO (with a 2-day lag) was significantly associated with ED visits for OM among females for warm, cold, and all seasons (Table 4). NO_2_ was also significantly associated with ED visits for OM in girls with a 2-day lag in all and warm seasons but not in cold seasons. An IQR increase in PM_10_ (4-day lag) was also associated with ED visits for OM in girls. Neither SO_2_ nor O_3_ was significantly associated with ED visits in females.

We performed calculations for multiple and cumulative exposure and presented illustrative results in the Supplemental Material (doi:10.1289/ehp.0901675). We observed that the models including both CO and NO_2_ gave higher effect estimates for CO than did the single CO model, and also significant negative associations for NO_2_. CO and NO_2_ are negatively correlated with O_3_ (−0.55 and −0.53, respectively), so a negative association with NO_2_ may indicate a positive association with high O_3_ on a preceding day. However, the hypothesis did not substantiate the analysis results when we used combinations of pollutants’ lags instead of one common lag. We concluded that the models where the two collinear pollutants encounter are unreliable.

PM_2.5_ and PM_10_ were also highly correlated (0.85) and as concurrent predictors gave unreliable results. PM showed only weak association with OM, also in multipollutant models. Adding one type of PM to models did not significantly affect the associations with the gaseous pollutants [see Supplemental Material, Table 4 (doi:10.1289/ehp.0901675)].

With those exceptions, the multipollutant model produced results agreeing with the results obtained with one-pollutant models. We did not perform separate residual analyses.

It appeared that most associations between the cumulative exposure and the health outcome were stronger and showed linear dependence on the dose accumulation period (the more days of accumulation, the higher the estimated odds). At the same time, some associations may disappear (e.g., O_3_ association with OM in male patients in warm season). We explained the vanishing of correlation when exposure accumulates as follows: Real health impacts caused by acute short-term excesses of exposure cannot be predicted by the accumulated exposure. Averaging the pollutant levels over few days (when building the cumulative exposure) flattens the excesses and scrambles the link between an excess and the issuing cases. In most cases we found that the seasonal and sex-related patterns in associations with cumulative exposures were comparable with the patterns we observed in analysis of lagged 1-day exposures [see Supplemental Material (doi:10.1289/ehp.0901675)].

## Discussion

In this study, we observed significant associations between ambient air pollutants and ED visits for OM in 1- to 3-year-old children in Edmonton, Alberta, Canada. This association was most notable during warm weather months. To our knowledge, we are the first to report significant associations between air pollutants and ED visits for OM in North America. Our study included > 14,000 ED visits for OM, making it larger than all previously published studies combined. In addition, the case-crossover study design used in our study, in which each child served as his or her own control, reduced or eliminated potential confounding effects of socioeconomic status, which may be associated with crowded living conditions and air pollution exposures.

OM exerts an enormous economic burden on the health care system, with estimated per episode costs ranging from $108 to $1,300 in the United States (Schwartz and Gates 2003). The costs and utility of observation and routine antibiotic treatment options for children with acute OM are in the range from $132 to $157 in the United States (Coco 2007). It is a common childhood illness that is one of the most frequent reasons for medical visits and antibiotic prescriptions (Rovers 2008).

The U.S. Surgeon General (U.S. Department of Health and Human Services 1986) and the National Research Council (1996) have confirmed a link between OM and environmental tobacco smoke. There are several proposed pathophysiological mechanisms to explain this association. First, pollutants can disrupt mucociliary clearance and eventually cause Eustachian tube dysfunction (Bluestone and Klein 1983). Second, reduced mucociliary clearance may result in a greater predisposition to upper respiratory viral illnesses and thus a resultant OM (Heikkinen et al. 1999). Third, pollutants may result in adenoidal hyperplasia, resulting in narrowing of the Eustachian tubes. Lastly, pollutants might directly cause mucosal swelling of the Eustachian tube (Ohashi et al. 1989a, 1989b). Whatever the mechanism, it is certainly plausible that that these same mechanisms could be attributed to traffic-related pollutants as well.

Prior research suggests that not all ambient air pollutants contribute to OM. From three repeated cross-sectional studies in East Germany, Heinrich et al. (2002) concluded that the adjusted time prevalence of OM was not associated with SO_2_ or total suspended PM. We also observed that IQR increases in SO_2_ levels were not significantly associated with ED visits for OM. It is possible that an effect of SO_2_ on OM would require a higher threshold level of exposure than we observed in Edmonton (Table 1). A small cross-sectional study of 393 11- to 13-year-old Brazilian children showed a positive association between OM prevalence and the mean level of SO_2_ (Ribeiro and Cardoso 2003), and it is likely that SO_2_ exposures were higher in São Paulo than in Edmonton or that SO_2_ was associated with other air pollutants.

PM_10_ and PM_2.5_ data were available for less time than for the other pollutants in our analysis; PM_10_ was measured since January 1994 and PM_2.5_ since April 1998. Although we observed significant associations with PM_10_ during the warm months only, the smaller power of this analysis might explain why other significant associations were not noted.

In general, our results indicated stronger associations when we modeled pollutants using a 2-day lag rather than longer or shorter lag times. This could be consistent with an effect of pollutant-induced mucosal swelling of the Eustachian tubes after a viral infection has already been established. Typically, a viral-mediated OM requires several days of nasal congestion before development of otalgia (ear pain). A lag of 2 days would not allow sufficient time for toxin-related injury to precede viral invasion and resulting otalgia (Corbeel 2007).

The above-mentioned argument also might account for the observed seasonal variation in the association. In the pediatric population, acute OM has a much higher incidence in winter than in summer, explained by the far greater incidence of respiratory viral illness in the winter. Our observed stronger association with pollutants in the warm weather months reinforces our hypothesis that pollutants are associated with OM. Edmonton’s climate demonstrates seasonal extremes: winters are cold (average high of −12°C in January) and the daylight hours are short (< 7.5 hr on the winter solstice), in contrast to warm summers with > 17 hr of daylight. This results in far greater indoor time for children in winter than in summer, which likely reduces the contribution of pollutant-related toxins on cases of OM. This might also explain why PM was significantly correlated in the summer months only.

In our study, we also analyzed potential associations between air pollutants and OM based on sex. We chose to perform this analysis because there was limited evidence to suggest that males might be biologically more susceptible than females to air pollution: in a French study, Granados-Canal et al. (2005) observed that adult males required more hospitalization for respiratory diseases after air pollution exposure than did females. Our data seem to indicate that seasonality has a stronger influence than does sex on OM with air pollution exposure (with warm seasons having a greater effect than cold seasons). In addition, different pollutants were more strongly associated with one sex: CO with a slightly stronger and prolonged lag association only in boys during warm seasons, and NO_2_ and PM_10_ with significant associations only in girls. It remains to be seen whether these observed variations are attributable to differences in biological susceptibility to specific pollutants between boys and girls, to other potential differences between sexes (e.g., amount of exposure to air pollutants, differing susceptibility to viruses, varying parental approaches to illness based on sex), or to chance variation.

In any study such as this, multiple comparisons are a concern. When so many analyses are undertaken, there are obviously going to be some statistically significant associations that are due to random variation or chance. We stratified on sex and season and focused our attention on patterns in the associations. For example, an IQR increase in O_3_ was significantly associated with ED visits for OM among boys, for all seasons and warm seasons. Also our analysis with subgrouped data restricted to the first and unique visits show the same pattern of responses [see Supplemental Material (doi:10.1289/ehp.0901675)].

### Limitations and strengths

One limitation of the study is the imprecise definition of the hazard period and the eventual ED diagnosis. We do not have data that elucidate individuals’ symptom durations before ED diagnosis, so we cannot determine when ED visits occurred in relation to the onset of symptoms. Although such data would not have changed the diagnosis of OM or the association with pollutant exposure, they might have assisted in further clarification of the lag-time relationship between pollutant exposure and symptom onset.

It is important to comment on why we chose our age range. The American Academy of Pediatrics and the American Academy of Family Physicians developed a joint clinical practice in 2004 for the management of OM. It recommendations include an “observation option” for the management of OM for children beyond the toddler years (except for severe cases) (American Academy of Pediatrics Subcommittee on Management of Acute Otitis Media 2004). Therefore, the most clinically significant patients are younger children; older preteens and teens are often managed with pain control only. Also, because our data did not allow for month resolution of age, we did not include children < 1 year of age. Because the anatomy and physiology of a neonate’s Eustachian tube greatly differ from those of an infant just < 1 year of age, it would be difficult to generalize our proposed disease model to this young age. Finally, although our age constraint includes the age with the peak incidence of OM (1–2 years of age) (Daly and Giebink 2000), it would have been interesting to analyze the relationship between OM and air pollutants for children 0–36 months of age using monthly age resolution.

Another potential limitation is that we averaged global exposure estimates across the three monitoring stations for most pollutants. These estimates did not reflect actual local exposures, which may have varied because of locally intensive motor vehicle traffic and other potential sources of pollution. We were unable to perform calculations based on the monitoring station closest to the child’s residence because we did not have complete postal code data on all the patients.

We also considered the potential misclassification of exposure and its effect on the estimates. The error in estimating personal exposure from three fixed-site monitor stations would tend to reduce the probability of detecting an effect and, in most cases, bias air pollution–OM correlations toward the null (Zeger et al. 2000). Fixed-site station measurement has been shown to correlate with both personal exposure and indoor exposure.

This is the first North American study to examine OM and air pollution levels, and it is also the largest study to date on OM and air pollution, doubling the existing body of evidence. By implementing a case-crossover design, we were able to reduce or eliminate both known and unknown confounders by cases serving as their own controls. This is an ideal study format for short-term exposures.

We collected data from five hospitals throughout this city of > 1 million people over 10 years, and we were able to examine environmental data for lags up to 4 days. We also performed subgroup analyses based on seasonality and sex. Prior research has been unable to definitely confirm an association between OM and air pollutants, largely because of the small sample sizes and insufficient power of the previous studies. We recognize that the regular care provider will diagnose most cases of OM. Because we did not have data for these visits, we used the five EDs across Edmonton as a proxy for the overall burden of the disease.

We did not collect data on smokers in the home, socioeconomic status, access to primary care, proximity to major roadways, time spent outdoors, or number of prior OM diagnoses. Although confounding by these variables would have been mitigated by the fact that patients acted as their own controls, future research might address interactions between air pollution and these factors.

Given the universal health care system in Canada, in which nearly all patients have a regular health-care provider, we would stipulate that there is in fact no major difference in the patient population that presents to the ED versus to the general practitioner for care. Patients will often to present to the ED out of convenience (after hours or weekend), and thus we believe these results can be generalized to the population at large.

## Conclusions

The results support the hypothesis that ED visits for OM are associated with ambient air pollution. Variation occurs between warm and cold weather periods in Edmonton, Alberta, Canada. The potential exists to reduce cases of OM in children by implementing policies to reduce the production of and therefore exposure to ambient air pollutants.

## Figures and Tables

**Table 1:** Daily average concentrations of the ambient air pollutants and meteorological factors, Edmonton, Alberta, Canada (1 April 1992–31 March 2002; 3,652 days).

Variable	Mean		All months	SD (all months)	Quartile (all months)		IQR
	Warm months (April–September)	Cold months (October–March)			First	Third	
CO (ppm)	0.5	0.9	0.7	0.4	0.4	0.8	0.4
NO_2_ (ppb)	16.5	27.2	21.9	9.4	14.7	27.6	12.8
SO_2_ (ppb)	2.1	3.1	2.6	1.8	1.3	3.5	2.3
O_3_ (ppb)	23.4	13.8	18.6	9.3	11.3	25.2	14.0
PM_10_ (μg/m^3^)	24.0	21.1	22.6	13.1	13.3	28.3	15.0
PM_2.5_ (μg/m^3^)	8.7	8.3	8.5	6.2	4.6	10.9	6.2
Temperature (°C)	13.1	−5.3	3.9	11.9	−4.0	13.9	17.9
Humidity (%)	62.9	69.1	66.0	13.6	57.1	75.6	18.5

First and third quartiles are 25th and 75th percentiles.

**Table 2:** Associations between pollutants and OM based on lag times (days): all patients [OR (95% CI)].

Pollutant	Lag	All months	Warm months	Cold months
CO	0	0.99 (0.97–1.01)	1.02 (0.95–1.10)	0.99 (0.96–1.01)
	1	0.99 (0.97–1.01)	1.08 (1.00–1.17)*	0.98 (0.96–1.00)
	2	1.03 (1.00–1.05)*	1.14 (1.06–1.23)*	1.02 (0.99–1.04)
	3	1.01 (0.99–1.03)	1.08 (1.00–1.16)*	1.00 (0.98–1.02)
	4	1.01 (0.99–1.03)	1.06 (0.99–1.15)	1.00 (0.98–1.02)

NO_2_	0	0.99 (0.96–1.02)	0.98 (0.90–1.06)	0.99 (0.96–1.03)
	1	0.99 (0.96–1.02)	1.03 (0.95–1.11)	0.98 (0.94–1.01)
	2	1.05 (1.01–1.08)*	1.10 (1.02–1.19)*	1.03 (1.00–1.07)*
	3	1.01 (0.98–1.04)	1.08 (1.00–1.17)*	0.99 (0.96–1.03)
	4	1.00 (0.97–1.03)	1.03 (0.95–1.12)	0.99 (0.96–1.03)

SO_2_	0	0.98 (0.95–1.00)	0.97 (0.93–1.02)	0.98 (0.95–1.01)
	1	0.99 (0.96–1.01)	0.99 (0.95–1.04)	0.99 (0.96–1.02)
	2	0.98 (0.96–1.01)	1.00 (0.95–1.04)	0.98 (0.95–1.01)
	3	0.99 (0.97–1.02)	1.03 (0.98–1.08)	0.97 (0.94–1.00)
	4	1.00 (0.98–1.03)	1.02 (0.98–1.07)	0.99 (0.96–1.02)

O_3_	0	1.04 (1.00–1.09)*	1.07 (0.99–1.16)	1.04 (0.98–1.10)
	1	1.04 (1.00–1.09)*	1.01 (0.93–1.09)	1.07 (1.01–1.14)*
	2	0.97 (0.92–1.01)	0.97 (0.90–1.06)	0.98 (0.92–1.03)
	3	1.02 (0.98–1.07)	1.02 (0.94–1.11)	1.04 (0.99–1.10)
	4	1.03 (0.98–1.07)	1.03 (0.95–1.12)	1.04 (0.98–1.10)

PM_10_	0	0.99 (0.96–1.03)	1.03 (0.98–1.08)	0.98 (0.94–1.02)
	1	0.99 (0.96–1.02)	1.03 (0.98–1.08)	0.97 (0.93–1.01)
	2	1.02 (0.99–1.05)	1.05 (1.00–1.10)*	1.01 (0.97–1.05)
	3	0.99 (0.96–1.02)	1.04 (0.99–1.09)	0.98 (0.95–1.02)
	4	1.01 (0.98–1.04)	1.05 (1.00–1.10)*	1.00 (0.96–1.04)

PM_2.5_	0	1.01 (0.97–1.04)	1.02 (0.97–1.08)	1.01 (0.96–1.06)
	1	0.98 (0.95–1.02)	1.03 (0.97–1.09)	0.97 (0.92–1.02)
	2	0.99 (0.95–1.02)	1.02 (0.96–1.08)	0.98 (0.93–1.03)
	3	0.96 (0.93–1.00)	0.99 (0.93–1.05)	0.97 (0.92–1.02)
	4	1.00 (0.96–1.03)	1.03 (0.98–1.09)	0.98 (0.93–1.03)

The results are reported for the IQRs listed in Table 1 and are lagged for temperature and humidity. Warm months are April–September; cold months are October–March.

* *p* < 0.05.

**Table 3:** Associations between pollutants and OM based on lag times (days): male patients [OR (95% CI)].

Pollutant	Lag	All months	Warm months	Cold months
CO	0	0.99 (0.96–1.02)	1.07 (0.97–1.17)	0.98 (0.95–1.01)
	1	0.99 (0.96–1.01)	1.13 (1.02–1.25)*	0.97 (0.94–1.00)
	2	1.02 (0.99–1.04)	1.17 (1.06–1.29)*	1.00 (0.97–1.03)
	3	1.00 (0.97–1.03)	1.08 (0.97–1.19)	0.99 (0.96–1.02)
	4	1.01 (0.98–1.04)	1.11 (1.01–1.23)*	1.00 (0.97–1.03)

NO_2_	0	0.99 (0.94–1.03)	0.97 (0.88–1.08)	0.99 (0.94–1.04)
	1	0.97 (0.93–1.01)	0.99 (0.89–1.10)	0.96 (0.92–1.01)
	2	1.03 (0.99–1.08)	1.04 (0.94–1.15)	1.03 (0.98–1.08)
	3	1.00 (0.96–1.04)	1.04 (0.94–1.16)	0.99 (0.94–1.04)
	4	1.00 (0.95–1.04)	1.04 (0.94–1.16)	0.99 (0.94–1.04)

SO_2_	0	0.98 (0.95–1.02)	0.97 (0.91–1.03)	0.99 (0.94–1.03)
	1	0.98 (0.95–1.02)	0.99 (0.93–1.05)	0.98 (0.94–1.02)
	2	0.98 (0.95–1.01)	0.97 (0.91–1.03)	0.98 (0.94–1.02)
	3	0.98 (0.95–1.02)	1.04 (0.98–1.11)	0.95 (0.92–0.99)*
	4	1.01 (0.97–1.04)	1.04 (0.98–1.11)	0.99 (0.95–1.03)

O_3_	0	1.09 (1.03–1.16)*	1.11 (1.00–1.24)*	1.09 (1.01–1.18)*
	1	1.06 (0.99–1.12)	0.97 (0.87–1.08)	1.12 (1.03–1.21)*
	2	0.99 (0.93–1.05)	0.97 (0.87–1.08)	1.02 (0.94–1.10)
	3	1.05 (0.99–1.12)	1.05 (0.94–1.17)	1.07 (0.99–1.15)
	4	1.02 (0.96–1.09)	0.98 (0.88–1.09)	1.06 (0.98–1.14)

PM_10_	0	1.02 (0.97–1.06)	1.04 (0.97–1.10)	1.00 (0.95–1.06)
	1	0.98 (0.95–1.03)	1.04 (0.97–1.11)	0.95 (0.90–1.01)
	2	1.01 (0.97–1.05)	1.05 (0.98–1.12)	1.00 (0.95–1.05)
	3	0.98 (0.95–1.02)	1.04 (0.97–1.11)	0.97 (0.92–1.02)
	4	1.00 (0.96–1.04)	1.03 (0.97–1.10)	1.00 (0.95–1.05)

PM_2.5_	0	1.03 (0.98–1.08)	1.04 (0.97–1.12)	1.04 (0.97–1.11)
	1	1.00 (0.95–1.05)	1.07 (0.99–1.15)	0.98 (0.91–1.04)
	2	0.97 (0.93–1.02)	1.01 (0.93–1.10)	0.97 (0.91–1.03)
	3	0.95 (0.90–0.99)*	0.96 (0.88–1.05)	0.95 (0.89–1.01)
	4	0.98 (0.93–1.02)	1.00 (0.93–1.08)	0.96 (0.90–1.03)

The results are reported for the IQRs listed in Table 1 and are lagged for temperature and humidity. Warm months are April–September; cold months are October–March.

* *p* < 0.05.

**Table 4:** Associations between pollutants and OM based on lag times (days): female patients [OR (95% CI)].

Pollutant	Lag	All months	Warm months	Cold months
CO	0	0.99 (0.96–1.02)	0.96 (0.85–1.08)	0.99 (0.95–1.02)
	1	1.00 (0.97–1.03)	1.02 (0.91–1.15)	0.99 (0.96–1.03)
	2	1.04 (1.01–1.07)*	1.12 (1.00–1.25)*	1.03 (1.00–1.06)*
	3	1.02 (0.98–1.05)	1.08 (0.96–1.21)	1.01 (0.98–1.04)
	4	1.01 (0.98–1.04)	1.00 (0.89–1.13)	1.00 (0.97–1.04)

NO_2_	0	1.00 (0.95–1.05)	0.99 (0.88–1.12)	1.00 (0.94–1.05)
	1	1.01 (0.96–1.06)	1.08 (0.96–1.21)	0.99 (0.94–1.04)
	2	1.06 (1.01–1.11)*	1.20 (1.06–1.34)*	1.04 (0.99–1.09)
	3	1.02 (0.97–1.07)	1.14 (1.02–1.29)*	0.99 (0.94–1.05)
	4	1.00 (0.95–1.05)	1.02 (0.91–1.15)	1.00 (0.95–1.05)

SO_2_	0	0.97 (0.93–1.01)	0.97 (0.90–1.04)	0.97 (0.92–1.01)
	1	1.00 (0.96–1.04)	1.00 (0.93–1.07)	1.00 (0.95–1.04)
	2	0.99 (0.95–1.03)	1.04 (0.97–1.11)	0.97 (0.93–1.01)
	3	1.00 (0.96–1.04)	1.01 (0.95–1.09)	0.99 (0.95–1.04)
	4	1.00 (0.96–1.03)	1.00 (0.93–1.08)	0.99 (0.95–1.04)

O_3_	0	0.99 (0.92–1.06)	1.02 (0.90–1.16)	0.98 (0.90–1.07)
	1	1.03 (0.96–1.10)	1.06 (0.94–1.20)	1.02 (0.94–1.11)
	2	0.94 (0.88–1.01)	0.98 (0.86–1.11)	0.93 (0.85–1.01)
	3	1.00 (0.93–1.07)	0.99 (0.87–1.12)	1.02 (0.94–1.11)
	4	1.03 (0.96–1.10)	1.10 (0.97–1.25)	1.02 (0.93–1.10)

PM_10_	0	0.97 (0.92–1.02)	1.02 (0.94–1.09)	0.95 (0.89–1.01)
	1	0.99 (0.95–1.04)	1.02 (0.95–1.10)	0.99 (0.93–1.05)
	2	1.02 (0.98–1.07)	1.05 (0.98–1.12)	1.01 (0.96–1.08)
	3	1.00 (0.96–1.05)	1.03 (0.96–1.11)	1.00 (0.95–1.06)
	4	1.02 (0.98–1.07)	1.07 (1.00–1.15)*	1.00 (0.95–1.06)

PM_2.5_	0	0.97 (0.92–1.03)	0.99 (0.90–1.08)	0.98 (0.91–1.06)
	1	0.96 (0.91–1.01)	0.98 (0.89–1.07)	0.96 (0.89–1.04)
	2	1.00 (0.95–1.06)	1.03 (0.96–1.10)	1.00 (0.92–1.07)
	3	0.99 (0.94–1.04)	1.02 (0.94–1.11)	1.00 (0.93–1.07)
	4	1.02 (0.97–1.07)	1.07 (0.99–1.16)	1.00 (0.93–1.07)

The results are reported for the IQRs listed in Table 1 and are lagged for temperature and humidity. Warm months are April–September; cold months are October–March.

* *p* < 0.05.
